# Biomarkers in Human Peripheral Blood Mononuclear Cells: The State of the Art in Amyotrophic Lateral Sclerosis

**DOI:** 10.3390/ijms23052580

**Published:** 2022-02-25

**Authors:** Orietta Pansarasa, Maria Garofalo, Eveljn Scarian, Francesca Dragoni, Jessica Garau, Rosalinda Di Gerlando, Luca Diamanti, Matteo Bordoni, Stella Gagliardi

**Affiliations:** 1 IRCCS Mondino Foundation, 27100 Pavia, Italy; orietta.pansarasa@mondino.it (O.P.); maria.garofalo@mondino.it (M.G.); eveljn.scarian@mondino.it (E.S.); francesca.dragoni@mondino.it (F.D.); jessica.garau@mondino.it (J.G.); rosalinda.digerlando01@universitadipavia.it (R.D.G.); luca.diamanti@mondino.it (L.D.); stella.gagliardi@mondino.it (S.G.); 2 Department of Brain and Behavioral Sciences, University of Pavia, Via Forlanini 6, 27100 Pavia, Italy; 3 Department of Biology and Biotechnology “L. Spallanzani”, University of Pavia, 27100 Pavia, Italy

**Keywords:** amyotrophic lateral sclerosis, biomarkers, peripheral blood mononuclear cells, RNA metabolism, protein alterations

## Abstract

Amyotrophic lateral sclerosis (ALS) is a devastating neurodegenerative disease, characterized by the progressive loss of lower motor neurons, weakness and muscle atrophy. ALS lacks an effective cure and diagnosis is often made by exclusion. Thus, it is imperative to search for biomarkers. Biomarkers can help in understanding ALS pathomechanisms, identification of targets for treatment and development of effective therapies. Peripheral blood mononuclear cells (PBMCs) represent a valid source for biomarkers compared to cerebrospinal fluid, as they are simple to collect, and to plasma, because of the possibility of detecting lower expressed proteins. They are a reliable model for patients’ stratification. This review provides an overview on PBMCs as a potential source of biomarkers in ALS. We focused on altered RNA metabolism (coding/non-coding RNA), including RNA processing, mRNA stabilization, transport and translation regulation. We addressed protein abnormalities (aggregation, misfolding and modifications); specifically, we highlighted that SOD1 appears to be the most characterizing protein in ALS. Finally, we emphasized the correlation between biological parameters and disease phenotypes, as regards prognosis, severity and clinical features. In conclusion, even though further studies are needed to standardize the use of PBMCs as a tool for biomarker investigation, they represent a promising approach in ALS research.

## 1. Introduction

### 1.1. Amyotrophic Lateral Sclerosis 

Neurodegenerative diseases (NDDs) are a broad group of debilitating pathologies that affect specific types of neural cells, leading to their death. Because of the increasing average age of the population, these age-related disorders are becoming more prevalent. They are different in their manifestations, due to the distinct types of affected cells and the variability of the pathophysiology. Nevertheless, they share common features: they can manifest in two main forms, sporadic and familiar, that may or may not be characterized by specific gene mutations, and they all lead to the accumulation of disease-specific proteins [1,2].

Amyotrophic lateral sclerosis (ALS) is one of the most diffuse NDDs. It affects upper and lower motor neurons, in the cortex, brainstem and spinal cord, causing weakness, muscle atrophy and spasticity. The consequent paralysis leads to death within two to five years after onset of symptoms [3]. Jean-Martin Charcot, who performed autopsies on patients who manifested weakness and atrophy, first described ALS in 1869. He correlated “brownish-gray streak marks” in the lateral column of the spinal cord with the spasticity of patients, and the lesions with the muscle wasting or amyotrophy. Many epidemiological investigations have been conducted on ALS, especially in Europe. A recent meta-analysis review concluded that the prevalence rates and the incidence rates were 6.22 and 2.31, respectively, for Europe, with an onset peak at 65 years. These same rates were, respectively, 5.20 and 2.35 for North America, 3.41 and 1.25 for Latin America, 3.01 and 0.93 for Asian countries, excluding Japan, and 7.96 and 1.76 for Japan [4,5]. Although the first definition focused mainly on motor symptoms, it is now evident that ALS is a multidimensional disease. Many patients develop cognitive and/or behavioral impairment, similar to frontotemporal dementia (FTD), in the so-called amyotrophic lateral sclerosis-frontotemporal spectrum disorder (ALS-FTSD) [6]. To date, although many studies have focused on ALS pathogenesis, Riluzole [7], a glutamate antagonism, is the only approved therapy in Europe.

As with other NDDs, ALS presents in two forms: the sporadic one accounts for 90% of the cases, whereas 10% of ALS patients have at least one family member in the familiar history, with an autosomal dominant inheritance in most of these cases. Familial cases are clinically indistinguishable from sporadic cases. Moreover, both forms can carry gene mutations and many unknown genetic factors may be present in sporadic cases without a genetic diagnosis [8,9]. A recent study demonstrated that the heritability of ALS was between 43% and 62%, with the highest values in mother–daughter pairings [10]. Regardless of the sporadic or familial form, it has been demonstrated that ALS patients carry a genetic risk from birth, which, interplaying with environmental factors, leads to the manifestation of the pathology [11,12]. Moreover, ALS can be classified by the site of onset; one-third of patients have a bulbar onset, which causes primary dysarthria and dysphagia, often associated with emotional symptoms, whereas 60% of the cases have a limb onset, which can be manifested in the upper or lower motor neurons [4].

Despite the different subtypes of ALS pathology, it can be caused by mutations in different genes. In addition to most common genes, such as SOD1, TARDBP, FUS/TLS and C9orf72, rarer genes were recently discovered, thanks to the progression of diagnostic technologies, i.e., VCP, OPT, UBQLN2 and ATXN2. The first gene discovered and determined to be involved in ALS pathology was superoxide dismutase 1 (SOD1), a metalloenzyme, which converts intracellular superoxide free radicals (O_2_-) into hydrogen peroxide (H_2_O_2_) and O_2_ [13]. The toxic function of this protein had been associated with oxidative stress, inflammation, mitochondrial toxicity and protein relocalization and aggregation [14,15,16]. TARDBP encodes the protein TDP-43, which, moving between nucleus and cytoplasm, regulates many pathways, such as RNA processing, transcription, translation and splicing, by binding the RNA itself [17]. In 2006, TDP-43 was found for the first time as the major component of ubiquitinated inclusions in ALS-FTD patients [18,19]. Mutations in TARDBP can cause TDP-43 mislocalization in the cytoplasm, its ubiquitination and hyper-phosphorylation, its truncation in toxic fragments and aggregation. Consequences can include impairment in endocytosis [20,21], mitochondrial toxicity [22,23,24] and, consequently, oxidative stress [25,26]. Curiously, mutations in the VCP gene, involved in both ALS and FTD, manifest ubiquitin and TDP-43 inclusions, and are grouped in the so-called proteinopathies [27]. Moreover, mutations in the FUS/TLS gene manifest FUS-immunoreactive aggregates, often positive to TDP-43, p62 and ubiquitin [28,29]. Finally, C9orf72 was the most recent gene found to be involved in ALS and FTD pathology [30,31]. The expansion of the hexanucleotide GGGGCC may lead to two different consequences: loss or toxic gain of function of C9orf72. An arbitrary cut-off of 30 repeats is used in most studies as a pathological repeat length threshold. The histopathological characteristics of ALS patients carrying this mutation are, in this case, TDP-43 inclusions, RNA foci comprising C9orf72 repeat RNA and dipeptide repeat proteins [32]. 

Finally, different single nucleotide polymorphisms (SNPs) were associated with ALS. Yet, in 2009, Sha and colleagues identified three SNPs involved in two two-locus combinations in sporadic ALS patients. Thanks to the advancements in GWAS technology, many ALS-associated SNPs are now known [33,34] and, more recently, Zhang and colleagues found a correlation between CpG-SNPs and ALS age of onset [35]. 

### 1.2. Peripheral Blood Mononuclear Cells Biomarkers

Biomarkers refer to a wide subcategory of medical and/or biological signs that can be measured objectively, accurately and reproducibly. They are sorted into [36]:

(i) diagnostic biomarkers, used to detect the presence of the disease or to identify subjects with a disease subtype;
(ii) predictive biomarkers, used to identify which treatment a patient or a group of patients will respond to;
(iii) prognostic biomarkers, used to predict disease progression.

Among the biological specimens employed in the field of biomarker discovery, special attention has been paid to blood-derived samples. Blood collection is simple and inexpensive; thus, it is a good source of biomarkers. Moreover, blood can be easily fractionated into its different components, i.e., plasma (or serum), buffy coat, containing white blood cells (WBCs) and platelets, and red blood cells (RBCs). Plasma, serum and peripheral blood mononuclear cells (PBMCs), obtained from buffy coat, are the most frequently used blood fractions in biomarker discovery research [37]. Many studies revealed that PBMCs, including T and B cells, natural killer cells and monocytes, could recapitulate the conditions of the surrounding tissue, thus, providing a suitable source of biomarkers. Additionally, PBMCs can be source of miRNA, mRNA, circRNA and methylation markers [38]. Moreover, PBMCs provide two further advantages: they can be kept in culture and they allow us to perform stimulation experiments, as demonstrated by Zondler and colleagues in 2016 [39].

Considering that ALS lacks effective treatment, and that life expectancy is, on average, only three years, it is imperative to shorten diagnosis and to initiate therapies promptly. In this perspective, biomarkers may be the key. Biomarkers can be a tool to sustain early diagnosis and predict prognosis, to stratify patients and to better understand ALS pathomechanisms, identify targets for treatment, and develop new and effective therapies. To date, knowledge of biomarkers of ALS is limited and no valid biomarkers for ALS are available, even if several ALS-related biological biomarkers have been detailed [40]. Interestingly, biomarkers from blood samples are receiving increased attention, mainly because they are an effective intermediary between central and peripheral damage in ALS [14,40,41].

However, in 2011, Nardo and colleagues described a strategy for the identification of PBMCs biomarkers, in order to study NDDs. They developed a panel of 14 validated proteins, distinguishing ALS patients with low disease severity from healthy individuals. In particular, they demonstrated that the levels of peptidyl-prolyl cis-trans isomerase A (PPIA), heat shock cognate protein 71 kDa (HSC70), heterogeneous nuclear ribonucleoprotein A2/B1 (hnRNPA2B1) and TDP-43 significantly differed between ALS patients and healthy controls [42]. More recently, the same group established a controlled procedure for the analysis of PBMCs markers in an enlarged cohort of sporadic ALS subjects and controls, confirming previous findings. Moreover, they identified PIA as a disease modifier with important prognostic potential [43]. 

Furthermore, Leoni and colleagues performed a cross-phenotype investigation in PBMCs and plasma, identifying other ALS-associated biomarker candidates, such as myosin-9, fructose-bisphosphate aldolase and plectin. Moreover, functional analysis showed an enhancement in pathways involved in immune system regulation [44]. 

### 1.3. White Cells Subpopulations and ALS

The immune system has emerged as an important player in NDDs such as ALS. Many studies have revealed aberrant immune responses in affected patients and animal models. White cells include lymphocytes, monocytes and granulocytes (neutrophils, eosinophils and basophils).

A first study, on 284 ALS patients and 217 age-matched controls, found a reduction in CD4+ T lymphocytes and circulating immune complexes (CICs) and an increase in component C3. Moreover, patients with severe or moderate impairment had a higher CD4+ T cell percentage [45]. 

In 2017, a study on 119 ALS patients and 35 healthy controls revealed that changes in neutrophil and CD4 T-cell number correlated with disease progression, with an increase in these cells in affected subjects [46]. In the same year, a study clustered 80 ALS patients in two distinct immune profiles. Subjects with an abnormal immune profile were younger, often with a familial form of ALS, and survived longer than patients more similar to healthy controls [47]. 

In 2020, McGill and colleagues studied leukocytes from 23 healthy controls and 48 ALS patients to measure leukocyte subpopulation alterations. They discovered an increase in the ratio of classical to non-classical monocytes in patients, compared to controls, and a correlation between greater disease severity and a reduction in non-classical monocytes. Moreover, they noted an association between myeloid markers’ expression, such as CD16 and HLA-DR, and ALS clinical features [48]. 

Finally, a recent study found a reduction in peripheral monocyte subsets, an increase in peripheral and intrathecal activation of CD4+ and CD8+ T cells and a decrease in intrathecal levels of immune-regulatory CD56 bright natural killer cells in ALS patients [49]. These results confirmed a previous study, which found a drastic reduction in regulatory T cells in ALS patients and a correlation between their number and the disease progression [50]. Moreover, it was found that self-reactive CD8+ T lymphocytes exerted a cytotoxic function in ALS mice [51]. 

These changes in white blood cell populations could also be found in other diseases, such as cancer, acute and chronic inflammation [52,53,54]. These studies suggested that when combined with each other, white cell subpopulations could be a promising source of biomarkers for the study of NDDs. 

## 2. Altered RNA Expression in Peripheral Blood of ALS Patients

### 2.1. RNA Metabolism Involvement in ALS Pathogenesis

The alteration of gene expression has been observed in ALS patients and models [55,56]; thus, RNA metabolism has emerged as a central process in disease etiology. Moreover, ALS causative mutations in RNA-binding proteins (RBPs), such as TARDBP (TDP-43) and FUS, have further corroborated the disease-specific perturbations in RNA processing [57,58] at gene transcription, mRNA stabilization, and transport and translation regulation. 

Approximately 97% of patients with Familial ALS (FALS) and Sporadic ALS (SALS) are positive for TDP-43 inclusions in the motor cortex and spinal cord, thereby establishing TDP-43 as a major protein signature for disease [8]. TDP-43 mRNA expression was found to be enhanced by 1.5–1.8-fold change in PBMCs from patients affected by ALS disease [59]. TDP-43 has been shown to play a role in RNA metabolism, including RNA transcription, alternative splicing, pre-microRNA processing, RNA transport and messenger RNA stability. It has demonstrated the ability to bind multiple mRNAs to regulate their splicing and to repress the splicing of cryptic exons [8], leading to nonsense-mediated decay (NMD) of the associated mRNA. Furthermore, TDP-43 has a role in mRNA stability and/or transport, since it is able to bind to the 3′ UTR of numerous mRNAs. It was reported that TDP-43 is necessary for regulating the expression of 239 mRNAs, whose resulting proteins are involved in synaptic functions and in the regulation of neurotransmitter processes [60]. Additionally, FUS can bind 3′ UTRs, regulating the stability of hundreds of transcripts, promoting their stabilization or destabilization [61]. FUS mutants have been shown to induce the upregulation of mRNA NMD machinery, resulting in a decrease in transcript stability and consequent reduction in translation [62].

TDP-43 and FUS also play a role in the biogenesis of miRNAs, endogenous small non-coding RNAs that recognize target mRNAs through base-pairing interactions and perform gene silencing. TDP-43, in particular, was shown to associate with proteins involved in the cytoplasmic cleavage of pre-miRNA, mediated by the DICER enzyme. Indeed, dysregulation of miRNAs has been observed in ALS, including miRNAs essential for motor neuron development and maintenance, axonal growth and synaptic transmission. Thus, alterations in these miRNAs may contribute to ALS pathogenesis [56]. In turn, miRNAs are targets of other non-coding RNAs, such as circular RNAs (circRNAs) and long non-coding RNAs (lncRNAs) [63]. CircRNAs are the product of pre-mRNA back-splicing and are resistant to RNA exonucleases, conferring stability in cells [64], while lncRNAs are transcripts of more than 200 nucleotides that interact with and regulate the behavior of proteins and/or other RNA molecules [65]. These classes of non-coding RNAs compete with other RNA species, for binding both RBPs or miRNAs [66], thus, creating a tightly controlled regulation network. Regulation of circRNAs-related splicing by FUS in human-induced pluripotent stem cell-derived motor neurons (MNs) carrying the FUS^P525L^ mutation was demonstrated and a circRNA-dependent regulatory network in MNs was described, thus, providing interesting understanding of the FUS-associated ALS pathogenic mechanism [67]. Additionally, the association of lncRNAs with ALS was explored [68], highlighting the involvement of several lncRNAs in ALS molecular mechanisms [69,70,71].

Interestingly, mRNA of SOD1, one of the ALS-causative genes, was found to be deregulated (overexpressed) in the spinal cord, brain stem and PBMCs obtained from SALS patients [72]. Moreover, mutant SOD1 (mutSOD1) gains RNA binding activities, thus, perturbing the turnover of the target mRNAs [73]. The acquisition of toxic and abnormal protein–RNA interaction ability may be caused by conformational changes due to mutation in this gene, since the wtSOD1 protein cannot bind RNAs because of the lack of a canonical RNA binding motif [73].

Hexanucleotide expansions in the *C9orf72* gene have been associated to ALS. A 50% reduction in C9ORF72 transcript was observed in patients with expansions [74], and it was hypothesized that expanded RNA forms pathogenic foci that trap one or more RNA binding protein(s). This results in RNA toxicity and loss of function of specific RBPs [75]. 

Studies based on RNA deep sequencing have pointed out the differential expression of several coding and non-coding RNAs, resulting in a negative regulation of the RNA transcription process and transcription factors activity [76], as well as aberrant alternative splicing [77] and, therefore, RNA processing. Consequently, the study and the understanding of RNA metabolism alterations in ALS could provide new insights on this fatal disease and perhaps lead to the discovery of possible prognostic biomarkers and/or therapeutic targets. 

In this section, we reported the most promising differentially expressed RNAs, found in the peripheral blood (PB) of ALS patients, to be used as peripheral biomarkers for both diagnosis and monitoring of the disease. 

### 2.2. Deregulated mRNA in ALS Patients’ Peripheral Blood

ALS has been recognized as a multi-systemic disease, where pathological processes do not only extend in motor neurons [78,79,80,81]. Peculiar features of ALS were also observed in PBMCs [14,42,76]. 

PBMCs from 74 SALS patients were screened through quantitative PCR (qPCR) and western blot (WB) for evaluating the expression of motor proteins involved in the anterograde and retrograde intracellular transport. The authors found that levels of KIF5C and DCTN1 were altered in patients compared to controls, thus, identifying putative useful biomarkers [82]. Moreover, they also compared SALS patients with patients of ALS-mimicking disorder, finding KIF1B expression as discriminant for the two groups of subjects [82]. Another study, involving 50 Indian SALS patients, investigated the expression of VEGF-A and CCL2, proinflammatories involved in neuroprotection molecules, in PBMCs through qPCR. Gupta and collaborators found that VEGF-A was upregulated when comparing patients to controls, while both VEGF-A and CCL2 upregulation was observed when comparing patients with respiratory dysfunction to patients without respiratory dysfunction [83]. In 2018, we published a whole transcriptome study of SALS/FALS PBMCs [76]; the two groups of ALS patients showed different gene expression patterns. Only one transcript involved in autophagy-related pathway [84], TPCN1, was common between SALS and FALS.

Similar studies have been conducted on peripheral blood leukocytes (PBL). One of these started from results obtained in human mesenchymal stem cells (hMSC), derived from the bone marrow of ALS patients, where a decrease in the expression of Cytoplasmic FMR Interacting Protein 2 (CyFIP2) and Retinoblastoma (Rb) Binding Protein 9 (RbBP9) was observed. Thus, the expression of these two genes was also tested in PBLs isolated from 17 SALS patients and a similar trend was found in non-neural tissues [85]. In addition, Vrabec and colleagues observed the overexpression of two genes linked to apoptosis and neuronal differentiation, apoptosis-associated tyrosine kinase (AKKT) and GTPase dynamin 2 (DNM2), in PBLs isolated from 84 SALS patients [86].

Interestingly, in the PB of SALS patients, the expression of genes known to be associated with FALS/SALS cases has been investigated. In fact, PARK7, C9orf72, ALS2, MATR3, SPG11, ATXN2 were confirmed to be dysregulated compared to healthy controls [87]. Three splicing-related genes were recently identified in a transcriptome study, conducted on 397 ALS patients and 645 control subjects. HNRNPUL1, SNRPN, and YTHDC1 were differentially expressed in PB samples in a heterogeneous ALS cohort [88].

Eventually, alteration of the gene expression through NGS was analyzed in a subpopulation of leukocytes and monocytes. In these cells, boosters of inflammatory response, ALS samples demonstrated a strong inflammation-related gene expression profile, where IL1B, IL8, FOSB, CXCL1 and CXCL2 expression was found to be significantly altered. Moreover, a greater proinflammatory signature in rapidly progressing patients was observed, compared to slowly progressing patients [89].

### 2.3. Deregulated microRNAs in ALS Patients’ Peripheral Blood

As revised by Ravnik-Glavač and Glavač [90], a plethora of potential circulating microRNAs biomarkers were identified in ALS patients’ biofluids (e.g., plasma, sera, extracellular vesicles and blood). In addition, Vijayakumar and collaborators conducted a literature search in order to extrapolate dysregulated microRNAs across different independent studies—mainly those involved in molecular processes typical of ALS pathogenesis [91]—thus, reducing the bottleneck of reliable biomarkers. Our aim was to provide information on microRNAs solely deregulated in blood cellular populations. 

Blood leukocytes isolated from SALS patients were characterized by the differential expression of several microRNAs, such as miR-9, miR-338, miR-638, miR-663a, miR-124a, miR-143, miR-132, miR-206, and let-7b, hsa-miR-451, miR-1275, hsa-miR-328-5P, miR-665, miR-183, miR-193b, miR-451 and miR-3935 [86,92,93,94]. Meanwhile, in peripheral blood samples, deregulated microRNAs included let-7a-5p, let-7d-5p, let-7f-5p, let-7g-5p, let-7i-5p, miR-103a-3p, miR-106b-3p, miR-128-3p, miR-130a-3p, miR-130b-3p, miR-144-5p, miR-148a-3p, miR-148b-3p, miR-15a-5p, miR-15b-5p, miR-151a-5p, miR-151b, miR-16-5p, miR-182-5p, miR-183-5p, miR-186-5p, miR-22-3p, miR-221-3p, miR-223-3p, miR-23a-3p, miR-26a-5p, miR-26b-5p, miR-27b-3p, miR-28-3p, miR-30b-5p, miR-30c-5p, miR-342-3p, miR-425-5p, miR-451a, miR-532-5p, miR-550a-3p, miR-584-5p and miR-93-5p [87].

Some of these microRNAs may be considered most promising [90] for their presence in multiple biomarker search analysis and for their biological relevance. In fact, let-7, miR-451 and miR-125a/b have been linked to neuroinflammation and microglia activation [95,96,97], while miR-338-3p and miR-183 have been linked to neuronal development [98,99]. Mir-338p has also been indirectly related to glutamate level maintenance [100]. Many studies have been conducted concerning the role of miR-206 in ALS pathogenesis. Apparently, the involvement of mir-206 was not strictly related to ALS onset, but rather to protective mechanisms activated during the first stages of disease, mostly related to promotion of the re-innervation of muscles by suppressing muscular HDAC4 protein levels [101,102].

### 2.4. Other Candidate Noncoding RNAs as ALS Biomarker in Peripheral Blood

In our study from 2018 [76], we also quantified the expression of lncRNAs in ALS patients’ PBMCs. In our SALS cohort, a total of 293 lncRNAs were differentially expressed. The great majority were uncharacterized antisense lncRNAs, but we also found interesting transcripts, such as ZEB1-AS and ZBTB11-AS1. Moreover, we found MYC-induced noncoding RNA (MINCR) downregulated. This lncRNA has been associated to various cancer types [103,104,105,106,107,108,109], as its upregulation has been linked to poor prognosis. In our cohort of SALS patients, MINCR was downregulated and its ambivalent behavior in different diseases could be used as a biomarker and/or therapeutic target [110]. 

Moreover, circRNA alterations in ALS patients’ leukocytes were identified. Those identified as potential blood-based biomarkers were: hsa_circ_0023919, hsa_circ_0063411 and hsa_circ_0088036, as of ALS [111].

## 3. Protein Expression in PBMCs of ALS Patients

Many neurodegenerative diseases, including ALS, are considered proteinopathies, usually represented by the accumulation of protein aggregates [112]. Moreover, protein accumulation can be exploited for the discovery of new biomarkers, represented by evaluating the protein expression level in cerebrospinal fluid (CSF), plasma and PBMCs of ALS patients [42]. Thus, the characterization of protein alteration in ALS patients’ PBMCs represents a starting point for the discovery of novel biomarkers useful for diagnosis, patients’ stratification and disease progression. 

SOD1 was the first gene identified in FALS and is involved in a great number of pathophysiological mechanisms [13,113]. Some mechanisms of disease implicated with SOD1, in both FALS and SALS, are protein misfolding, proteasome impairment, oxidative stress, oligodendrocyte degeneration and mitochondrial dysfunction [113]. Indeed, markers of oxidative damage were found in the central tissue (i.e., motor cortex and spinal cord) of both FALS and SALS patients [114]. In a previous study by our group, we found an over-expression of SOD1 mRNA in patients’ PBMCs but a decrease in the protein expression level when measured in the soluble fraction of cell lysates [72,115]. The authors hypothesized that this discrepancy was due to relocalization into different subcellular fractions, such as to the nucleus, or to aggregation/precipitation in the insoluble fraction [14]. By confocal microscopy and immunoblotting, they found an increased intensity of SOD1 signal in the nuclear compartment in a subset of SALS patients, while cytoplasmic SOD1 aggregates were found in the other ALS subjects. Moreover, they found a higher presence of detergent-resistant SOD1 in patients with aggregated cytoplasmic SOD1 [14]. In following works, the group found that the relocalization of SOD1 was caused by post-transcriptional modification (phosphorylation of SOD1 threonine and serine), and that nuclear SOD1 protects DNA from oxidative stress damage through the HSP70 pathway [15,116]. 

Mutations in the TARDBP and FUS gene are mainly associated with ALS, and both are involved in RNA processing. Both proteins tend to accumulate in ALS patients’ cells and tissues [117]. De Marco and colleagues reported that TDP-43 was expressed at about the same levels in whole cell lysates of ALS patients’ PBMCs, relatives and controls. On the other hand, they also found accumulation in the cytoplasmic fraction of TDP-43+ patients, TDP-43- patients and relatives of mutated patients [118]. These findings suggested that PBMCs could be informative of TDP-43 mislocalization and, thus, could be used as biomarkers of the pathology [118]. In contrast, no data were present in the literature on expression of the FUS protein in the PBMCs of ALS patients. An increase in protein aggregation was found only in lymphoblastoid cell lines (LCLs) and induced pluripotent stem cells (iPSCs), both derived from PBMCs [119,120]. 

The most common genetic abnormality in ALS is the expansion of GGGGCC (G_4_C_2_)_n_, translated through a repeat associated non-ATG (RAN) mechanism [121,122], resulting in the production of dipeptide repeat (DPR) proteins, i.e., Poly-GA, Poly-GP, Poly-GR, Poly-PA, and Poly-PR [123,124]. DPR proteins are considered a good biomarker for diagnosis, but also for evaluated pharmacodynamics in clinical trial. In particular, Gendron and colleagues reported PBMCs as a good source of DPR proteins in the blood samples of ALS patients [125].

Except for SOD1, the other genetic abnormalities can also be found in FTD, which shares several pathological characteristics with ALS. The two diseases cannot be discriminated using peripheral biomarkers, because they are distinguished only by the region they affect in the central nervous system (CNS), symptoms, and onset age of the disease [126].

Many other proteins are involved in ALS [112]. For example, Kuźma-Kozakiewicz and colleagues focused their work on the expression of motor proteins involved in both the anterograde and the retrograde transport in PBMCs obtained from SALS patients, healthy control subjects and other neurological disease(s) patients [82]. The authors reported a decrease in KIF5C and KIFC3 expression and an increase in DCTN1 expression in SALS compared to healthy subjects. Moreover, KIF1B, DCTN1 and DCTN3 were statistically significant in classic ALS but not in other neurological diseases [82]. Another important pathological mechanism in ALS is protein homeostasis. Vat and colleagues focused their attention on this topic and found an accumulation of low molecular weight ubiquitinated proteins in the PBMCs of SALS patients, suggesting the deregulation of proteasome pathways. In addition, they evaluated the conversion between LC3-I into LC3-II, reporting an increase in LC3-II/LC3-I ratio in the PBMCs of SALS patients, suggesting the involvement of autophagy pathways [127]. 

### Mass Spectometry Analysis in PBMCs of ALS Patients

Many other proteins are involved in ALS pathogenesis and can be used as biomarkers. Thus, mass spectrometry (MS) for proteomics analysis in ALS patients was exploited for the first time in both CSF and plasma, using matrix-assisted laser desorption ionization–time of flight and surface-enhanced laser desorption/ionization, to drive the identification of novel candidate disease biomarkers [128,129,130,131]. These studies opened promising pathways for biomarker investigation, but they had two limitations: CSF collections are not always well tolerated by patients, and highly abundant plasma proteins (albumin and IgGs, among others) limit the detection of the other proteins [44]. The use of PBMCs overcomes the issue of highly abundant plasma proteins, allowing the detection of less abundant proteins. Thus, PBMCs might represent a useful source of proteins for biomarker discovery using a proteomics approach with MS [44,132].

In 2009, Nardo and colleagues evaluated the nitrosylation of PBMCs protein with a proteomics approach. They found an increase in nitrosylation in SALS patients but, interestingly, they identified three major overnitrated proteins that overlapped between SALS patients and the rat model, i.e., actin, ATPase and vinculin. These proteins were reported to be nitrated more than three-fold in SALS patients with severe forms of the disease compared to healthy controls [133]. Moreover, they reported nitrosylation of vinculin and filamin-A in the PBMCs of SALS patients and rats, suggesting a change in the cytoskeletal properties of peripheral cells, influencing motility resistance to apoptosis [133]. The same group also analyzed 2D difference gel electrophoresis PBMCs of healthy controls and SALS patients with two levels of disease severity. They detected 129 protein spots, differentially expressed, that were analyzed in MS. They identified 71 candidate protein biomarkers implicated in energy metabolism, redox regulation, protein folding and degradation, cytoskeleton, inflammatory response, and DNA/RNA binding. Further validation allowed the identification of CALR, CLIC1, IRAK4, GSTO1 and CypA as ALS biomarkers, while CypA, TDP-43 and ERp57 as biomarkers for disease progression [42]. Filareti and colleagues used a similar approach, finding isomerase activity, protein folding, and response to stress among the most significantly enriched biological processes, identifying diverse protein, i.e., PPIA, HSP90, GRP78, ERp57, and DJ-1, as candidate phenotypic biomarkers [134]. In 2020, the same group studied a larger cohort of SALS patients and reported PPIA, hnRNPA2B1 and TDP-43 as the proteins most closely associated with SALS. In particular, they found that low PPIA soluble levels were correlated with earlier death, suggesting that PPIA could be a disease modifier with prognostic potential [43]. Leoni and colleagues conducted another important proteomics study. They performed conventional multiplex proteomic analysis of the PBMCs of both bulbar onset ALS (B-ALS) and limb-onset SALS (L-ALS). They characterized peptides expressed in PBMCs, deregulated in specific phenotypic variants of the disease, more significantly in B-ALS compared to L-ALS, providing a strong ground to develop biomarkers for clinical stratification in ALS. Indeed, principal component analysis (PCA) showed that the site of disease onset was probably the main contributor underlying biological differences across the two phenotypes. In particular, B-ALS clusters seemed to be more dispersed than the one seen in L-ALS, suggesting a higher degree of variability in B-ALS PBMCs [44]. Moreover, the group reported a deregulation of the proteins involved in immunoregulatory interactions between lymphoid and non-lymphoid cells, RHO GTPases and Sema4D signaling. The first pathway was extensively investigated in ALS to establish a way of immunomonitoring the disease [135]. The RHO GTPases pathway had a regulatory role in cytoskeletons formation, gene transcription, cell–cell adhesion, cell cycle progression, neuronal apoptosis and modulation of microglial activity [136,137]. Finally, the Sema4D signaling pathway was involved in neurite outgrowth and angiogenesis [138]. Thus, the authors concluded that their datasets had dissected many peripheral molecular changes that could be exploited in the future, as early diagnostic biomarkers, to differentiate diverse clinical phenotypes of ALS [44].

## 4. Biomarkers and Clinical Correlation

One of the main applications of biomarkers is their correlation with biological parameters and phenotypes, in terms of prognosis, severity and clinical features.

Peripheral biomarkers of ALS have been investigated for several years and correlations with inflammatory pathways in blood samples of ALS patients have emerged. Zhao and coauthors characterized peripheral monocytes of patients with ALS in terms of gene expression signature [89]. Their data showed a correlation between the number of proinflammatory deregulated genes and disease progression. In particular, a group of genes related to inflammatory response highly participated in immune response during fast progression and could contribute to accelerating disease progression. Additionally, it was demonstrated that, in the macrophages and monocytes of ALS patients, interleukin levels were high and correlated with disease burden, while TNFα protein was positively correlated with disease progression rates [139,140].

Regarding ALS survival and progression rates, microarray expression profiles in PBMCs showed that the RNA expression of genes related to inflammation was correlated with ALS clinical features [141].

## 5. Conclusions

Peripheral cells, such as PBMCs, seemed to share many pathological features, usually found in the central tissue. Indeed, many authors found disease-specific pathological alteration, not only in ALS, but also in other NDDs [142]. In particular, PBMCs can play a pivotal role in the study of NDDs, since the affected tissue is not easily accessible to evaluation. Moreover, PBMCs represent a better source for biomarker analysis, with respect to CSF, since they are easier to collect. PBMCs are also considered a better protein-based biomarker source, with respect to plasma, since they do not have plasma proteins that can disturb the detection of lower expressed proteins. Finally, they can be used to subdivide patients based on the specific molecular signature, thus, representing a reliable model for patient stratification. In conclusion, many other studies are needed in order to standardize the studies and the investigations on PBMCs, but they represent a promising model for these diseases. 
